# Immunotoxicity of Three Environmental Mycotoxins and Their Risks of Increasing Pathogen Infections

**DOI:** 10.3390/toxins15030187

**Published:** 2023-03-02

**Authors:** Yuhang Sun, Yuqi Song, Miao Long, Shuhua Yang

**Affiliations:** College of Animal Science & Veterinary Medicine, Shenyang Agricultural University, Shenyang 110866, China

**Keywords:** mycotoxins, aflatoxin B1, ochratoxin A, deoxynivalenol, bidirectional immunotoxicity, pathogen infections

## Abstract

Aflatoxin B1 (AFB1), ochratoxin A (OTA), and deoxynivalenol (DON) are the three mycotoxins that have received the most scholarly attention and have been tested most routinely in clinics. These mycotoxins not only suppress immune responses but also induce inflammation and even increase susceptibility to pathogens. Here, we comprehensively reviewed the determining factors for the bidirectional immunotoxicity of the three mycotoxins, their effects on pathogens, and their action mechanisms. The determining factors include mycotoxin exposure doses and times, as well as species, sex, and some immunologic stimulants. Moreover, mycotoxin exposure can affect the infection severity of some pathogens, including bacteria, viruses, and parasites. Their specific action mechanisms include three aspects: (1) mycotoxin exposure directly promotes the proliferation of pathogenic microorganisms; (2) mycotoxins produce toxicity, destroy the integrity of the mucosal barrier, and promote inflammatory response, thereby improving the susceptibility of the host; (3) mycotoxins reduce the activity of some specific immune cells and induce immune suppression, resulting in reduced host resistance. The present review will provide a scientific basis for the control of these three mycotoxins and also provide a reference for research on the causes of increased subclinical infections.

## 1. Introduction

Mycotoxins are the product of mold metabolism during growth and reproduction and often have a high level of toxicity and a low molecular weight. They are generally only created by a low number of molds: while one mold can result in the production of numerous mycotoxins, multiple molds can also produce a single mycotoxin. Aflatoxin B1 (AFB1), ochratoxin A (OTA), and deoxynivalenol (DON) are the three mycotoxins that have received the most attention from scholars and have been tested most routinely in clinics [1]. AFB1, OTA, and DON mycotoxins are produced by *Aspergillus flavus* (*A. flavus*) and *Aspergillus parasitica* (*A. parasitica*), *Aspergillus* (*A.*) and *Penicillium*, and *Fusarium*, respectively, and are widely found in moldy food, feed, and their raw materials, including peanut meal, soybean meal, and corn, especially in high-temperature and high-humidity areas. A survey reported by Bai’aoming Feed Additives (China) Co., Ltd. in 2021 showed that the positive rates of AFB1 and DON in new corn from eight major producing areas of China were 7–91% and 81–100%, respectively; the positive rate of AFB1 was 100% in peanut meal, and the positive rates of DON were 94% in bran meal, 83% in rice bran meal, and 100% in corn by-products; in addition, the positive rates of AFB1 and DON in commercial pig feed were 21% and 99%, respectively. OTA was also reported to be frequently detected in the above raw materials, and there was 50% OTA contamination in South Korea [2]. These three mycotoxins not only have hepatotoxicity, nephrotoxicity, and enterotoxicity but also have strong immunotoxicity, seriously impairing human and animal health. In addition, some reports showed that their combined toxicity was more severe than their individual toxicity [3,4].

It is traditionally believed that AFB1, OTA, and DON exposure can lead to immunosuppression in pigs at all ages. However, recent studies have shown that these three mycotoxins not only inhibit immunity but also induce inflammation [5,6,7]. However, their specific time, dose, and mechanism of action remain unclear. It is worth noting that exposure, even to low concentrations of AFB1, OTA, and DON, is unsafe as they induce inflammation, thereby increasing the risk of pathogen invasion [8,9,10]. At present, 37 countries all over the world have set limits for these three mycotoxins in food or cereals. The FDA and China’s Ministry of Agriculture stipulate that the limits of AFB1, OTA, and DON in cereals and cereal products (including corn, corn flour, and wheat) are 20 μg/kg, 50 μg/kg, and 1000 μg/kg, respectively. However, previous studies have shown that 750 to 1500 μg/kg DON can markedly promote the invasion and replication of porcine epidemic diarrhea virus [9,11]; 20 to 40 μg/kg AFB1 significantly promotes swine influenza virus infection and induces lung inflammation after 14 days of AFB1 exposure but promotes immunosuppression after 21 to 28 days [8,12]. Thus, it is important to know the determining factors of bidirectional immunotoxicity and its action mechanism and to summarize the influences of mycotoxins on pathogens and their mechanisms. The present paper will provide a scientific basis for the control of the above three mycotoxin contaminations and also provide a reference for research on the causes of increased subclinical infections.

## 2. Distribution and Characteristics of the Three Environmental Mycotoxins

### 2.1. AFB1

Aflatoxin, a low molecular secondary metabolite, is produced by *A. flavus* and *A. parasitica*. There are eighteen different varieties of aflatoxins, such as AFB1, AFB2, AFG1, AFG2, AFM1, and AFM2, of which AFB1 is the most important aflatoxin due to its toxicity and carcinogenic effects. AFB1 is widely distributed in moldy agricultural products, especially peanut meal and soybean meal (raw materials of animal feed), which seriously harm the health of livestock and poultry [13]. Many previous studies reported that AFB1 exposure can cause DNA damage, oxidative stress, and apoptosis and induce severe hepatotoxicity and nephrotoxicity [14,15,16].

### 2.2. OTA

After aflatoxin was discovered, ochratoxin ensured that people recognized mycotoxins again. Isocoumarin produces more than 20 chemicals, including ochratoxin, by cross-linking L-phenylalanine. OTA has attracted the attention of researchers due to its wide dissemination (moldy food and feed), serious toxicity, and significant impact on animals and agricultural products [13,17]. OTA is naturally produced by fungi such as *A. ocher*, *anthrax A.*, *A. niger*, and *Penicillium verrucosa*. OTA is toxic to livestock, and its primary target organ is the kidney. OTA can cause immunotoxicity, hepatotoxicity, apoptosis, decreased cell viability, and even affect oocyte maturation and embryonic development.

### 2.3. DON

Deoxyniniolenol, commonly known as DON, is a metabolite of *Fusarium* and is named for its ability to induce vomiting. DON is the most common contaminant in grains such as corn, wheat, and barley. When DON contaminates livestock and poultry feed, it is subsequently found in milk, meat, and eggs [13]. In animals, it causes organ damage and lipid accumulation in the liver, vomiting, anorexia, growth retardation, immunotoxicity, and impaired reproductive and developmental abilities. In addition, DON is cytotoxic to livestock and primarily attacks the gastrointestinal tract, especially the intestinal epithelial cells [18]. At the cellular and molecular levels, DON can induce apoptosis, oxidative stress, and genotoxicity and even affects the spindle morphology of porcine oocytes.

## 3. Bidirectional Immunotoxicity of the Three Mycotoxins, Determining Factors, and Their Action Mechanisms

### 3.1. Bidirectional Immunotoxicity of the Three Mycotoxins

**Figure 1:**
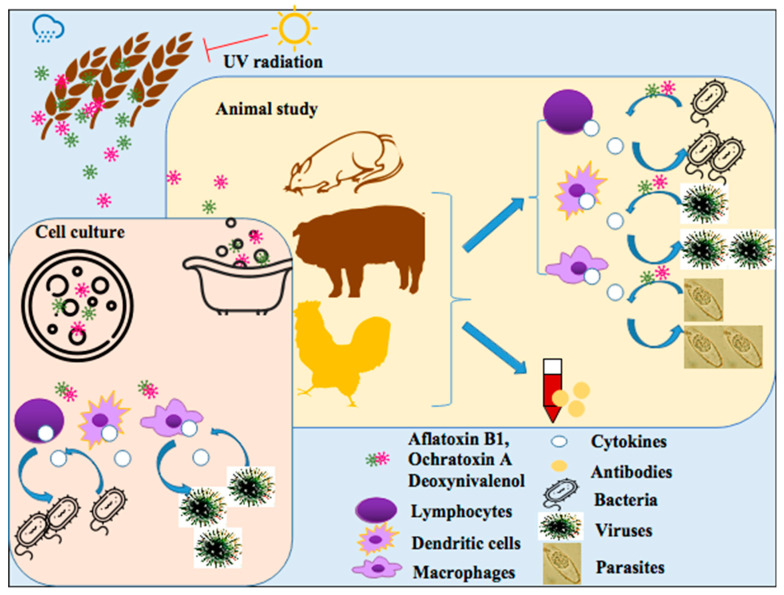
Immunotoxicity assessment studies on cultured cells in vitro and animals exposed to the three mycotoxins. These three mycotoxins work directly not only on cultured immune cells in vitro, including lymphocytes, dendritic cells, and macrophages, but also on animals, including mice, pigs, and chicks. They also work on primary cells isolated from these animals and up-regulate or down-regulate serum antibody levels and cytokine productions, thereby exerting immunotoxicity and even promoting pathogen infections.

Bidirectional immunotoxicity, also called two-way immunotoxicity, is defined as mycotoxins not only inhibiting immunity but also stimulating immunity and inducing inflammation [7]. It is traditionally believed that the immunotoxicity of mycotoxin is immunosuppression, but there are more and more reports on the inflammatory response induced by mycotoxin exposure. The bidirectional immunotoxicity of mycotoxins exhibits the toxic effect of mycotoxins on innate and adaptive immunities of animals (mice, pigs, and chicks) and cells, including affecting the proliferation, differentiation, or maturation of immune cells, such as lymphocytes, dendritic cells, and macrophages, cytokine production, antibody levels, and even increasing the susceptibility to pathogens (bacteria, viruses, and parasites) (Figure 1).

### 3.2. Determining Factors for Bidirectional Immunotoxicity

Immune suppression or promotion depends on the mycotoxin exposure dose and time, species, sex, as well as immunologic stimulants, and other factors (Table 1).

#### 3.2.1. Exposure Dose

A previous study indicated that low (0.01 μg/mL) and moderate levels (0.1 μg/mL) of AFB1 exposure could promote TLR4 and cytochrome P450 1A1 expressions [19]. In contrast, another study indicated that exposures to 4 to 8 μg/mL AFB1 markedly suppressed the proliferation of primary porcine splenocytes and decreased IL-2 production [21]. These two studies suggest that low-dose AFB1 exposure induces inflammatory responses, while high-level AFB1 exposure promotes immunosuppression.

There are only a few available studies on the bidirectional immunotoxicity of OTA. A previous study demonstrated that 0.5 to 4 μg/mL OTA exposure could suppress the proliferation of porcine primary splenocytes [22]. However, our previous study showed that 0.5 to 1.5 μg/mL OTA exposure could increase TNF-α production and up-regulate the TLR4-MyD88-NF-κB signal pathway [20].

DON also plays both immunostimulatory and immunosuppressive roles at different exposure doses: low doses of DON increase cytokine, chemokine, and inflammatory gene levels accompanied with immunostimulation, but elevated amounts of DON decrease cell proliferation, increase apoptosis and necrosis of immune cells with concomitant immune suppression and increase IgA secretion and susceptibilities to infections [29,30]. The previous report also proves that DON has two-way immune effects [5]: low doses of DON exposure enhance TNF-α and IL-6 expressions (pro-inflammatory cytokines) in vivo (750 μg/kg diet) and in vitro (1 and 2 μg/mL) and increase the chemotaxis and phagocytosis of pig alveolar macrophages while promoting macrophage polarization to M1. However, high doses of DON exposure enhance transforming growth factor beta and IL-10 (anti-inflammatory cytokines) expressions in vivo (3000 μg/kg diet) and in vitro (4, 6, and 8 μg/mL), suppress the chemotaxis and phagocytosis of pig alveolar macrophages, and promote macrophage polarization to M2.

#### 3.2.2. Exposure Time

The bidirectional immunotoxicity of the three mycotoxins is also significantly changed by their exposure time. Our previous study showed that short-term exposure (15 days; 8 h) to a given dose of AFB1 (40 µg/kg b.w.; 0.04 µg/mL) increased pro-inflammatory cytokine expressions; however, long-term exposure (18 and 21 days; 24 and 48 h) to AFB1 enhanced anti-inflammatory cytokine levels [8]. Correspondingly, long-term AFB1 exposure markedly reduced lymphocyte subsets and splenic and serum TNF-α, IL-2, IL-17, and interferon-γ productions and down-regulated the expressions of Th1, Th2, Th17, and Treg genes, thereby inducing immunosuppression [31].

Similarly, a recent study indicated that long-term instead of short-term OTA exposure was immunosuppressive: short-term OTA exposure (24 h) increased the pro-inflammatory cytokine expressions, migration, and phagocytosis of macrophages and promoted macrophage polarization to M1. However, long-term OTA exposure (72 h) increased anti-inflammatory cytokine expressions, decreased the phagocytosis and migration of macrophages and promoted macrophage switching from M1 to M2 [6]. The study supports the idea that exposure time will significantly influence the immunotoxicity of OTA.

Unfortunately, there have been no reports on the influence of exposure time on the bidirectional immunotoxicity of DON. Moreover, most previous studies reported the immunotoxicity of consecutive or repeated exposures to mycotoxins [6,8,31], with no reporting of a comparison of immunotoxicity between successive and discontinuous exposure. A single exposure (low and moderate levels) to mycotoxins may not threaten immunity and even may be beneficial, but further investigation is required.

#### 3.2.3. Species

Pigs are the most sensitive animal to the three mycotoxins, followed by humans and poultry, fish and shrimp, with ruminants being the least susceptible [7]. This order may vary slightly for a particular mycotoxin. The LD_50_ of AFB1 is 360, 1000, and 500,000 µg/mL for poultry, rodents, and ruminants, respectively. The maximum tolerance content of AFB1 is close to 385 µg/kg for pigs. A dose of 500 µg/kg AFB1 exposure induces immunotoxicity in juvenile Pacific white shrimp [23]. The bidirectional immunotoxicity of OTA has significant species-specific differences: the LD_50_ of OTA is 1000 to 6000, 2000 to 3000, and 48,000 to 58,000 µg/kg for pigs, rats, and mice, respectively, and 10 to 50 µg/mL OTA exposure plays various immunotoxic roles in various human cells [24]. For DON, pigs are also more sensitive than mice, poultry, and ruminants [25].

Unfortunately, there has been no comparative study on immunotoxicity among different animal species. We speculate that the order of the immunotoxicity sensitivity for different species of animals is the same as above, according to the currently available studies. However, the immunotoxicity concentration is much lower than above.

#### 3.2.4. Sex

To date, there are no reports comparing the influence of male and female animals on the immunotoxic outcomes of AFB1 and OTA. In contrast, the immunotoxicity of DON is widely believed to be sex-dependent. A previous study demonstrated that CD11b^+^ leukocyte numbers decreased in female instead of male mice fed DON relative to a control diet, suggesting that females could be more sensitive to DON than males [32]. A recent study also reported that sex hormones could influence the immunotoxicity of DON, and the innate and adaptive immunity of female mice was susceptible to DON [26]. However, other studies indicated that, after DON exposure, female mice produced higher levels of IgG and IgA, while males exhibited higher levels of IL-6 in the blood [33,34]. Therefore, the influence of sex on the bidirectional immunotoxicity of mycotoxins needs to be further investigated.

#### 3.2.5. Immunologic Stimulants

Immunologic stimulants, including lipopolysaccharide (LPS), phytohemagglutinin (PHA), concanavalin A (CoA), and pokeweed mitogen, are another determining factor affecting the bidirectional immunotoxicity of mycotoxins. Mycotoxins played immunosuppressive roles when stimulatory factors were present but immunostimulatory roles when they were absent [7]. A previous study showed that AFB1 had an immunosuppressive effect on the PHA-stimulated lymphocytes [27]. Without any immunologic stimulants, OTA exposure alone could increase TNF-α production, up-regulated TLR4, MyD88, and phosphorylated NF-κB p65 [20]. DON inhibited the LPS-induced NO and IFN-β secretions, resulting in its immunotoxic effects [28]. DON also significantly suppressed the up-regulation of maturation markers for dendritic cells, including CD86 and chemokine receptor 7 [35].

### 3.3. Mechanisms of the Bidirectional Immunotoxicity of the Three Mycotoxins

**Figure 2:**
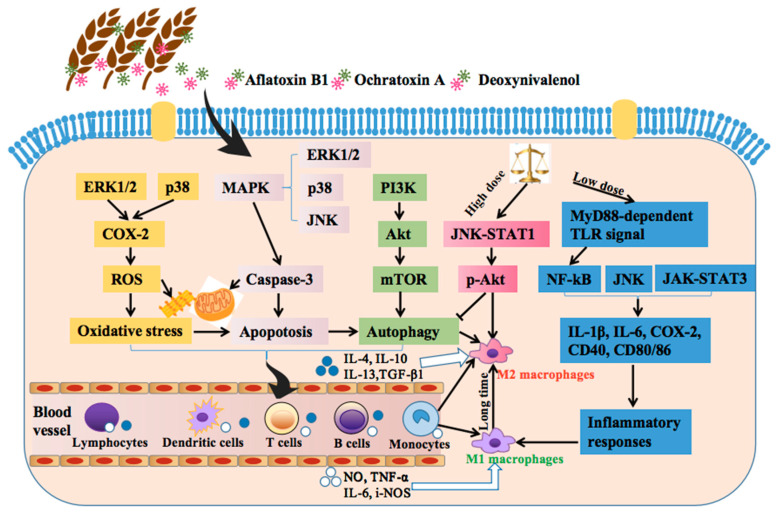
Mechanisms of action of the two-way immune effects of the three mycotoxins. M1 (immunostimulatory) and M2 (immunosuppressive) macrophages. Low-dose or short-term exposure to mycotoxins induces inflammation, but high-dose or long-term exposure to mycotoxins promotes immunosuppression.

The immunotoxic mechanisms of the three mycotoxins primarily participate in the oxidative stress, apoptosis, and autophagy of some immune cells and also regulate the immunity-related signals (Figure 2). These three mycotoxins can regulate some signal pathways, including extracellular signaling kinase (ERK) 1/2, P38, and mitogen-activated protein kinase (MAPK), thereby promoting the oxidative stress, apoptosis and autophagy of some immune cells. These immune cells include lymphocytes, dendritic cells, T cells, B cells, and monocytes, and they can secrete pro-inflammatory and anti-inflammatory cytokines, which can also promote the differentiation of monocytes into M1 and M2 macrophages, respectively. Moreover, these three mycotoxins can also directly activate the c-jun amino-terminal kinase (JNK)-STAT1 and MyD88-dependent TLR signal pathways, thus inducing immunosuppressive and inflammatory responses, respectively.

## 4. Effects of the Three Mycotoxins on Pathogen Infections and Their Action Mechanisms

**Figure 3:**
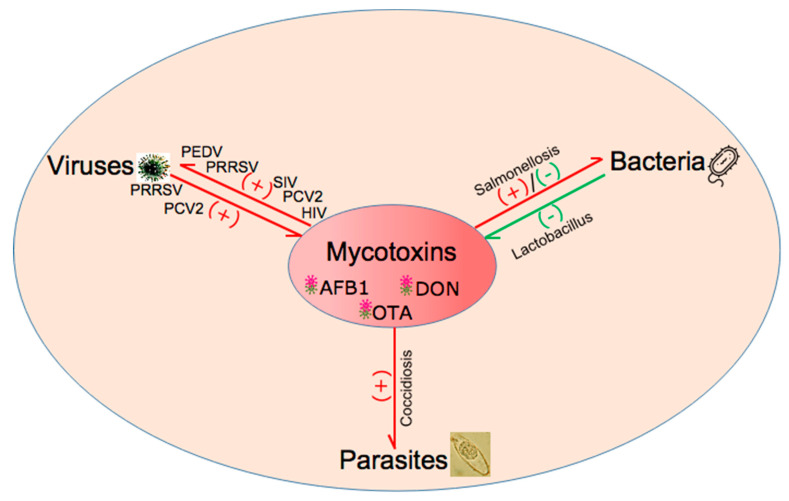
The interaction between the three mycotoxins and microorganisms, including viruses, bacteria, and parasites. These three mycotoxins can promote various types of virus replication, including porcine epidemic diarrhea virus (PEDV), porcine reproductive and respiratory syndrome virus (PRRSV), swine influenza virus (SIV), human immunodeficiency virus (HIV), and porcine circovirus type 2 (PCV2). In turn, PCV2 and PRRSV can aggravate the toxic effect of mycotoxins. The three mycotoxins can increase or reduce the number of bacteria, and some beneficial bacteria can protect the body against damage induced by mycotoxins. Mycotoxins also increase the proliferation of coccidiosis. (+) represents the promoted function; (−) represents the inhibitory function. Red and green lines represent harmful and beneficial functions, respectively.

Two decades ago, a review proposed that mycotoxins could increase the susceptibility to infectious diseases of farm animals, including chicks and pigs, and the severity of infections [36]. Subsequently, more and more studies on the interaction between mycotoxins and microorganisms, including viruses, bacteria, and parasites, have been reported. Of course, the effect could be positive or negative, and their interaction relationships are shown in Figure 3.

### 4.1. Effects of the Three Mycotoxins on Viral Infections

As reported in 2012, DON decreased the antibody titers of infectious bronchitis virus in broilers [37]. As reported in 2013, AFB1 could increase the antibody titers of the Newcastle disease virus and infectious bursal disease virus in broilers [38]. Jolly et al. reported that AFB1 could increase the viral load of HIV [39,40]. Similarly, another study in 2014 reported that DON could decrease the immune response against porcine reproductive and respiratory syndrome (PRRSV) and affect the course of infection of this virus in pigs [41]. Nevertheless, no data for the proliferation of microorganisms were reported in these studies. In contrast, Savard et al. demonstrated that DON significantly reduced the replication of PRRSV [42]. Moreover, Savard et al. also showed that low-dose DON promoted PCV2 replication [43].

In fact, these three mycotoxins indeed promote viral replication. AFB1 could promote swine influence virus (SIV) replication in vitro (Table 2) and in vivo [8,12,44,45]. By studying the results of several cell lines, it was shown that AFB1 might significantly accelerate SIV replication in vitro. In addition, in these studies, mice were also employed as a model to reveal that AFB1 can increase SIV reproduction in vivo. Meanwhile, AFB1 was also considered a critical factor for the transmission and pathogenesis of HIV [46] and acted with the hepatitis virus, promoting the development of hepatocellular carcinoma [47]. In addition, OTA promoted porcine circovirus type 2 (PCV2) replication [10,48,49,50]; DON promoted porcine epidemic diarrhea virus (PEDV) replication [11].

In turn, virus infection also aggravated the toxic effects of mycotoxins. As reported, PRRSV infection exacerbated the anorectic effects of high levels of DON exposure [41]; PCV2 infection aggravated OTA-induced nephrotoxicity [51]. Meanwhile, PCV2 and DON co-incubation promoted inflammatory responses [52].

### 4.2. Effects of the Three Mycotoxins on Bacterial Infections

Mycotoxin exposure affects the body’s gut microbiota. Gut microbiota refers to the microbial community in the intestinal tract that functions in a symbiotic manner, showing a dynamic structural balance, and its structure is affected by many factors. Gut microbiota is a link between external substances and host metabolism. There is a bidirectional relationship between gut microbiota and mycotoxins ingested by animals.

Usually, when animals ingest mycotoxin-contaminated feed, the gut barrier acts as a resistance. However, some mycotoxins still enter the gastrointestinal tract to exert virulence and even affect the composition and structure of intestinal microorganisms. For example, a moderate dose of AFB1 can adversely affect the intestinal barrier and increase gut permeability in broiler chickens [53]. OTA diet can decrease the diversity of gut microbiota in rats [54].

When the balance of intestinal flora is broken, exogenous pathogenic microorganisms adhere to the intestinal mucosa, causing a series of intestinal diseases, including diarrhea and even enteritis, thereby endangering the body’s health [55]. For example, AFB1 could increase *Escherichia coli* [45] and enhance the infected severity of *Salmonellosis* in chicken and Japanese quail. Meanwhile, aflatoxin increases the infected severity of experimental *Erysipelothrix rhusiopathiae* in swine. In addition, OTA could also increase the susceptibility of chickens to *Coccidiosis* and *Colibacillosis*. Stoev et al. reported that OTA contamination increased the susceptibility of swine to natural *Salmonella enterica*, *Brachyspira hyodysenteriae*, or *Campylobacter coli* infections [56].

On the other hand, mycotoxin exposure can also reduce the number of some intestinal flora, and even some beneficial bacteria can alleviate the toxicity of mycotoxins in turn. For example, *Lactobacillus* is a critical genus for detoxifying OTA in vivo [54]. Intestinal flora has been shown to mediate the protective effects of *Lactobacillus plantarum* on the apoptosis and intestinal inflammation of broilers exposed to DON [57].

### 4.3. Effects of the Three Mycotoxins on Parasitic Infections

There are few studies on the effect of mycotoxins on parasitic infection. The low-level ingestion of aflatoxin could increase the infected severity of *coccidiosis* in broilers [58]. AFB1 could also promote the rupture of toxoplasma cysts in previously infected mice, and the percentage of ruptured cysts increased from 15% to 56% after AFB1 exposure. Moreover, OTA was also proved to elevate the susceptibility of broilers to *coccidiosis*. A study indicated that OTA-treated chicks and turkeys experienced faster and more harmful *Eimeria acervulina* and *E. adenoeides* infections than the control animals [59]. DON had the potential to regulate immune responses after coccidial infection [60].

### 4.4. Mechanisms of the Three Mycotoxins Affecting Pathogen Infections

As outlined in the above discussion of the relationship between mycotoxins and bacteria, viruses, and parasites, we found that mycotoxin exposure can affect microorganism infections, and the specific mechanism of action includes three aspects: (1) mycotoxin exposure directly promotes the proliferation of pathogenic microorganisms; (2) mycotoxins produce toxicity, destroy the integrity of the mucosal barrier, and promote inflammatory response, thereby improving the susceptibility of the host; (3) mycotoxins reduce the activity of specific immune cells including macrophages to induce immune suppression, finally resulting in reduced host resistance.

#### 4.4.1. Mechanisms of the Three Mycotoxins Affecting Viral Infections

**Figure 4:**
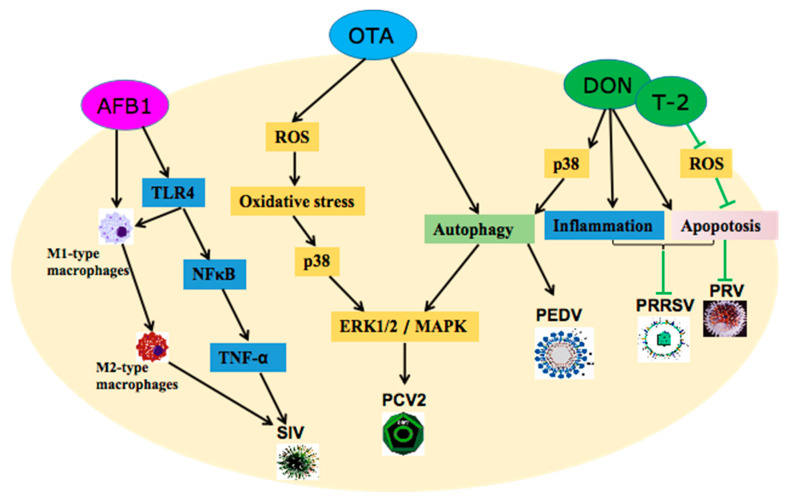
Mechanisms of the three mycotoxins affecting viral infections. Porcine epidemic diarrhea virus (PEDV), porcine reproductive and respiratory syndrome virus (PRRSV), swine influenza virus (SIV), human immunodeficiency virus (HIV), and porcine circovirus type 2 (PCV2).

Short-term exposure to AFB1 promoted SIV replication via up-regulating the TLR4-NFκB pathway, but long-term exposure to AFB1 increased SIV replication via promoting macrophages polarizing from M1 towards M2 due to excessive inflammatory responses [8,12]. OTA promoted PCV2 replication via up-regulating the p38/ERK1/2 MAPK pathway medicated by oxidative stress [48]. In addition, OTA has also been proven to increase PCV2 replication by inducing ROS-mediated autophagy [10]. Recently, DON (0.1, 0.5, and 1 µg/mL) was proven to increase the entry and replication of PEDV by inducing p38-mediated autophagy [9]. In contrast, DON (0.14 and 0.28 µg/mL) was proven to decrease PRRSV replication by promoting inflammation and accelerating apoptosis [42]. Similarly, low-concentration T-2 toxin (T2; T2 and DON belong to the trichothecene family, and are trichothecene A and B, respectively) could decrease the replication of pseudorabies virus (PRV) via down-regulating the oxidative stress- and apoptosis-related pathways [61]. The mechanism of mycotoxins affecting viral infections is summarized in Figure 4.

#### 4.4.2. Mechanisms of the Three Mycotoxins Affecting Bacterial Infections

Although many previous studies have confirmed that these three mycotoxins can promote bacterial proliferation, there are few studies on the mechanism of the mycotoxins affecting bacterial proliferation. On the contrary, there have been many recent studies on the mechanism of some beneficial bacteria reducing the toxicity of these mycotoxins. For example, *Lactobacillus plantarum* (*L. plantarum*) JM113 can alleviate DON-induced apoptosis and intestinal inflammation by improving bacterial community composition [57]. Some of them have also confirmed that large amounts of purification of specific critical proteins in beneficial bacteria can reduce the toxicity of the three mycotoxins and are expected to biodegrade mycotoxins [62]. For example, *Bacillus amyloliquefaciens 10* (B10) could be used as a feed additive to alleviate the AFB1-induced apoptosis, oxidative damage [63,64], and cecal inflammation in mice via modulating intestinal flora [65].

#### 4.4.3. Mechanism of Mycotoxins Affecting Parasitic Infections

There are few studies on the mechanism of mycotoxins affecting parasitic infections. For instance, aflatoxin can promote coccidial infection when it is co-infected with *E. tenella* [58]; DON can affect coccidial infection via recruiting T cells and macrophages to the jejunum [60].

## 5. Conclusions

In conclusion, the mycotoxins AFB1, OTA, and DON have bidirectional immunotoxicity: low-dose or short-term mycotoxin exposure induces inflammation, while high-dose or long-term mycotoxin exposure results in immunosuppression; when immunologic stimulants are present, the three mycotoxins mainly manifest anti-inflammatory effects. The bidirectional immunotoxicity mechanism of mycotoxins is involved in the oxidative stress, apoptosis, and autophagy of some immune cells and some immunity-related signals. Moreover, exposure to these mycotoxins can affect the infection severity of bacteria, viruses, and parasites. Their action mechanisms include three aspects: (1) mycotoxin exposure directly promotes the proliferation of pathogenic microorganisms; (2) mycotoxins produce toxicity, destroy the integrity of the mucosal barrier, and promote inflammatory response, thereby increasing the susceptibility of the host; (3) mycotoxins reduce the activity of some specific immune cells and induce immunosuppression, resulting in reduced host resistance.

## 6. Perspectives

Mycotoxin exposure is of great significance to microbial infection. It can directly affect the proliferation of microorganisms and can also promote the infection of pathogenic microorganisms by destroying the integrity of host mucosa, causing inflammation or immunosuppression. It may be difficult to eliminate the damage caused by mycotoxins completely. Nonetheless, it is essential to clarify the influences and mechanisms of mycotoxin exposure on microbial infections and increase people’s awareness that mycotoxin contamination may increase infectious diseases. Elucidating the impact of mycotoxin exposure on microbial infection will also be beneficial for studying the pathogenic mechanism of mycotoxin further and promoting the feasibility of prevention and control of mycotoxin contaminations to reduce its harm to the aquaculture industry.

## Figures and Tables

**Table 1:** Determining factors of the bidirectional immunotoxicity of the three mycotoxins.

Determining Factors	AFB1	OTA	DON
Exposure dose	(Low-dose exposure) inflammation [19]	(Low-dose exposure) inflammation [20]	(Low-dose exposure) inflammation [5]
	(High-dose exposure) immunosuppression [21]	(High-dose exposure) immunosuppression [22]	(High-dose exposure) immunosuppression [5]
Exposure time	(Short-term exposure) inflammation [8]	(Short-term exposure) inflammation [6]	-
	(Long-term exposure) immunosuppression [8]	(Long-term exposure) immunosuppression [6]	
Species	Pigs > ducklings > rats > sheep [23]	Pigs > rats > mice [24]	Pigs > mice > poultry > ruminants [25]
Sex	-	-	Female > male [26]
Immunologic stimulants	(With PHA) Immunosuppression [7,27]	-	(With LPS) Immunosuppression [28]
	(Without PHA) Inflammation [7]		(Without LPS) Inflammation [7]

AFB1: Aflatoxin B1; OTA: ochratoxin A; DON: deoxynivalenol; Immunologic stimulants, including lipopolysaccharide (LPS), phytohemagglutinin (PHA), concanavalin A (CoA), and pokeweed mitogen, are determining factor affecting the bidirectional immunotoxicity of mycotoxins.

**Table 2:** Effects of AFB1 exposure on SIV replication in multiple cell lines [12].

Concentration of AFB1 (μg/mL)	MDCK			A549			PAMs		
	Viral Titer	Viral M mRNA Levels	Viral NP Levels	Viral Titer	Viral M mRNA Levels	Viral NP Levels	Viral Titer	Viral M mRNA Levels	Viral NP Levels
0.01	↑	↑	↑	↑	↑	↑	↑	↑	↑
0.025	-	-	-	-	-	-	↑	↑	↑
0.05	↑	↑	↑	↑	↑	↑	↑	↑	↑
0.25	↑	↑	↑	↑	↑	↑	-	-	-

The effect of AFB1 on the replication of the H1N1 swine influenza virus (SIV) was studied by in vitro infection. Viral titer, M protein mRNA levels, and NP protein levels were detected to assess the level of H1N1 replication. AFB1: Aflatoxin B1; MDCK: Madin-Darby canine kidney cells; A549, human non-small cell lung cancer cells; PAMs: porcine alveolar macrophages.

## Data Availability

The data presented in this study are available in this article.
